# Development of a Psychosocial Intervention to Promote Treatment Adherence in Patients with Bipolar Disorder at Risk of Cardiovascular Disease

**DOI:** 10.3390/jcm10245890

**Published:** 2021-12-15

**Authors:** Sandra I. Ralat, Giselle Alicea-Cuprill, Yashira Arroyo, William Otero

**Affiliations:** 1 Department of Psychiatry, Medical Sciences Campus, University of Puerto Rico, San Juan, PR 00936, USA; 2 Clinical Psychology Program, San Juan Campus, Albizu University, San Juan, PR 00901, USA; gisi.marie@gmail.com (G.A.-C.); yarroyope88@gmail.com (Y.A.); wotero2465@hotmail.com (W.O.)

**Keywords:** psychosocial intervention, adherence, bipolar disorder, treatment, cardiovascular disease, risk factors

## Abstract

Nonadherence to treatment is a serious concern that affects the successful management of bipolar disorder (BD) patients. The aim of this study was to pilot test a psychosocial intervention (previously developed by this team) intended to increase adherence to medication and health behaviors targeting cardiovascular disease (CVD) risk factors in BD patients. An open, single-group design was used to assess the feasibility and acceptability of the intervention. The participants had BD, type I/II or unspecified, and CVD risk factors. Baseline demographic measures were taken. We also obtained preliminary effect sizes related to pre-post changes on measures of self-reported adherence to psychiatric medication, depressive and manic symptoms, and pharmacy records. At baseline, 29% of the participants reported recent adherence to psychiatric medications. A total of 71% of the participants completed the intervention. Pre-post improvements by medium and large effect sizes (Cohen’s *d* = 0.52–0.92) were seen in medication adherence, attitudes toward medication, and mania symptoms. The participants reported high levels of satisfaction with the intervention. A culturally sensitive psychosocial intervention for Puerto Rican BD patients who are at risk of CVD was found to be feasible and acceptable. Improvements in the key outcomes were seen in this small, preliminary study. Further research is needed with a larger sample size.

## 1. Introduction

Bipolar disorder (BD), formerly known as manic-depressive illness, is a psychiatric disease of major public health importance whose symptoms are severe and disabling; it provokes suffering in the patient as well as his or her family and loved ones and affects about 45 million people worldwide [1]. Individuals with BD experience extreme fluctuations in their moods, cycling between feeling elated and energized (manic episodes) to feeling sad, “down,” and/or hopeless (depressive episodes). [2]. There is also a euthymic episode in which the range of emotions extends neither to manic nor depressed but remains normal. The prevalence of BD is usually reported to be from 1% to 2%, but when we take into account subthreshold cases, the prevalence rate reaches as high as 6.4% [3]. After schizophrenia, BD is one of the costliest diseases in the United States. The healthcare costs associated with BD are high, but there are also other, less tangible costs, such as family burden and impaired health-related quality of life [4]. It is widely recognized that BD is associated with increased and premature mortality due to medical causes. Individuals with BD are vulnerable to a variety of physical conditions—hypertension, hyperlipidemia, type 2 diabetes, and metabolic syndrome, among others—and all are known risk factors for cardiovascular disease (CVD). Although CVD risk factors have been associated with psychiatric medications, several authors have found that CVD mortality was unusually high in BD patients prior to the advent of atypical antipsychotics as well as prior to the use of tricyclic antidepressants and/or lithium [5]. BD patients also have worse physical health and significantly lower life expectancies than the general population does [4,6].

Lack of medication adherence is a common but modifiable risk factor in BD patients [7]. Nonadherence to treatment is a serious concern due to the impulsive and high-risk nature of BD patients’ behaviors, which might provoke the adoption of unhealthy lifestyles (poor diet, cigarette smoking, physical inactivity, high levels of stress), themselves leading to an increase of CVD risk; working with BD patients is a significant challenge for the physician and healthcare systems [8]. Various authors have noted that Hispanics in the continental United States tend to be less adherent to their medication regimens than are their non-Hispanic white counterparts. Hispanic BD patients tend to be less treatment compliant with their other medical conditions as well. They have high prevalence rates of several illnesses that are also CVD risk factors (e.g., diabetes [9], hypertension, and hyperlipidemia) [4,10]. For all patients with psychiatric disorders, suboptimal adherence is a major barrier to favorable health outcomes and leads such nonadherent patients to experience significantly more hospitalizations, more absences from work, increased suicidal behavior, and poor adherence to medications prescribed for comorbid medical conditions [10].

In Puerto Rico (PR), there is no program for BD patients at risk of CVD that has proven to be effective in promoting adherence. In this study, adherence was seen as medication treatment compliance and the engagement of healthy behaviors (learned via a targeted strategic intervention program for patients with BD at risk of CVD). Family support is a relevant component in the process for adherence, and such support is considered a protective factor (against nonadherence) [10].

The aim of this study was to test a targeted intervention to promote treatment adherence in Hispanic BD patients at risk of CVD; said intervention was tested, initially, on patients living on the island of PR. The successful implementation of this protocol will result in the improvement of the psychiatric symptoms of our population of BD patients and will have a positive impact on those comorbid illnesses that these patients suffer and that also represent CVD risk factors. As many authors have stated, adherence is an issue of great importance for the successful treatment of BD, but there are few protocols targeting adherence in BD patients [7,11].

Our goals were to develop a psychosocial intervention program that would result in BD patients being able to significantly improve in terms of adhering to psychiatric medication and to pilot test the targeted intervention and determine its efficacy. Our objectives were that BD patients at risk of CVD show significant improvements in their adherence to psychiatric and CVD medications (primary outcome) based on pre- and post-intervention measures and that the use of the targeted psychosocial intervention program would result in significant improvements in the manic and depressive symptomatology and health behaviors of our participants (secondary outcome), which we would then determine using pre- and post-treatment measures.

## 2. Materials and Methods

### 2.1. Stage Ia—Development of the Psychosocial Intervention

The conceptual frame of this study was based on the stage model of behavioral therapy (SMBT) that was developed by Rounsaville, Carroll, and Onken [12]. There are three basic stages comprising studies that steadily evolve from feasibility (stage I) to efficacy (stage II) to transportability (stage III). In our study, we divided stage I into two substages.

The goal of this stage 1a was to draft the treatment protocol of a psychosocial intervention based on the input of the focus groups formed, in our earlier study, of BD patients at risk of CVD and thus create a manual [13]. We chose participants with BD because the literature reveals that Latinos with BD living in the continental United States are more likely to be nonadherent to psychiatric medication and to such medication as may be required for other medical conditions than are Euro-Americans [4,10].

The psychosocial intervention program that we developed includes different therapeutic techniques to modify patterns of behavior, emphasizing the psychological aspects of a person or group and considering the group’s situation from both a societal and a familial perspective [14]. This psychosocial intervention was developed to be culturally specific [15,16].

Twelve sessions were developed; each was divided into three modules for an optimal length of 90 min. This was a group therapy modality having one session per week. The first module included strategies for adherence and was divided into six sessions: identifying barriers to adherence, managing symptoms and stigma, establishing goals, managing medication, defining the role of the health provider, and determining the role of the family. The second module was composed of four sessions for the development of health behaviors, and the third module was the two last sessions, which covered, respectively, interpersonal relationships and support groups. In addition, we included a psychoeducational session in which a family member or significant other participated in the intervention. Through this manual, we based the intervention on the needs of BD patients at risk of CVD because of their lack of both adherence to medication and healthy behaviors. In the manual, we established the order of treatment procedures. In general, four approaches that have been demonstrated to be effective in the process of promoting adherence to treatment were used. These approaches consisted of (1) motivational interviewing to facilitate the development of life goals (including those of improving medication use and engaging in stimulating physical activities that promote healthy behaviors); (2) cognitive–behavioral techniques, both to overcome beliefs regarding medication and to help in the development of insight about the illness; (3) a family psychoeducation session for social–emotional support; and (4) symptom management. Each participant received a folder with the agenda of the sessions, brochures, and information related to each week’s session. See Table 1.

**Figure 1:**
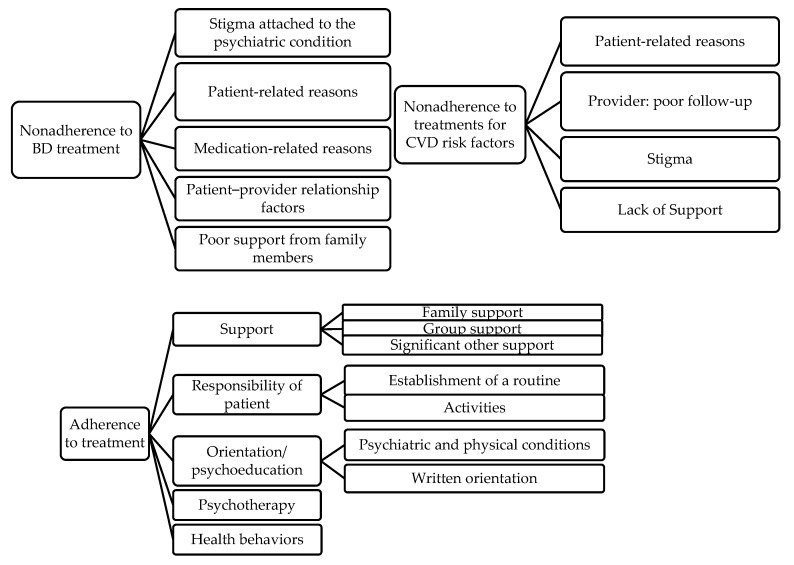
Barriers to adherence.

Figure 1 shows the barriers to and facilitators of adherence to BD and CVD medications as determined by an earlier study [13]. The topic and organization of the manual was discussed with several members of the team.

### 2.2. Stage Ib—Implementation of the Psychosocial Intervention

#### 2.2.1. Participants and Procedures

Stage Ib consisted of a pilot test the intervention that allowed us to make adjustments to any part of the treatment protocol and/or research method as might be necessary and assess the feasibility of said protocol. 

The sample included 24 patients with BD (types I, II, or unspecified) who were at risk of CVD and whose adherence to treatment was poor; all the patients were from the Mental Health and Substance Abuse Administration (ASSMCA, using its initials in Spanish) or the Community Mental Health Clinic at Albizu University. The sample consisted of men (8%) and women (92%) who were aged 25 to 60 years and were nonadherent to their psychiatric medication. These patients were at risk of CVD, having been diagnosed with hypertension, diabetes, obesity, and/or high cholesterol or triglycerides (at least one of the previous) and also having unhealthy lifestyles that included a poor diet, cigarette smoking, and/or physical inactivity. We excluded BD patients who had used illegal substances within the three months prior to the initiation of the pilot.

All the participants had bipolar disorder type I, II, and unspecified, which had been diagnosed by a psychiatrist. The CVD risk factors indicated by the participants had been diagnosed by physicians. ASSMCA has, among others, psychiatric and physician services; thus, the participants had all been diagnosed for several years and had prescriptions for their diagnoses, (confirmed with the pharmacy records obtained from the patients). The Community Mental Health Clinic at Albizu University provides psychological services to the community and receives patients with a diagnosis of BD.

The Institutional Review Board of the University of Puerto Rico Medical Sciences Campus approved this study. Clinical psychologists and/or social workers at the facilities (or supervised graduate students in psychology from Albizu University) invited qualified individuals to participate. The PI was notified to contact the potential candidates. Before a given individual’s enrollment in the study, he or she provided a signed consent form. All the subjects that signed the written informed consent answered a questionnaire that solicited sociodemographic and mental and physical health data. 

Stage Ib involved of an open, single-group design in which each subject was his or her own control. We assessed the feasibility and acceptability of the psychosocial intervention and also refined the manual based on patient feedback. The 24 participants were divided into three groups of eight, and all the participants received the same intervention. Pre- and post-tests were given to each participant at just two time points, the second being at week 13. The baseline assessment consisted of the administration of the questionnaire that gathered sociodemographic and health data. This questionnaire contained three questions about adherence to medication (self-report). The three questions that were included related to adherence behaviors (self-reported) and were as follows: (1) *How many times did you forget to take your medication, from last week until today?* (2) *How many times did you stop taking your medication, from last week until today?* and (3) *Do you have any problems taking your medication?* The Drug Attitude Inventory (DAI-10, Spanish version) was used to measure, in part, adherence/nonadherence to medication because a negative attitude toward medication is a major difficulty in the treatment of bipolar symptomatology. We also used the Spanish versions of the Young Mania Rating Scale (YMRS) and the Montgomery–Åsberg Depression Rating Scale (MADRS) for mood assessment; we used the Mini-Mental State Examination, second edition (MMSE-2), to rule out dementia and severe cognitive deterioration and administered the SF-36 Health Survey (SF-36) questionnaire [17,18,19,20,21].

The SF-36 is a 36-item health survey [21] that is used in clinical practice and research to evaluate nine health concepts. This is a multi-item scale, and the score for each is the weighted sum of the questions for that scale transformed to a scoring system that ranges from 1 to 100. Lower scores indicate relatively more disability or poorer health than do higher ones. The scales associated with physical health status were physical functioning (PF), role limitations linked to physical health (RP), and bodily pain (BP). The PF scale enquires into the current health of the participants, asking whether they have any limitations in terms of their abilities to exert themselves. The scale breaks the concept of “exertion” into three categories: heavy exertion (e.g., running, lifting heavy objects, playing sports), moderate exertion (e.g., moving a table, vacuuming), and mild exertion (e.g., picking up and carrying a full bag, climbing a single floor, kneeling). The RP scale asks about problems with work or other daily activities as a result of physical health. The BP scale assesses whether a given individual suffered any kind of limiting pain. We observed a decrease in the scores of that scale as well. Furthermore, there were three scales associated with the mental component of health status. The scales were role limitations due to emotional problems (RE), social functioning (SF), and emotional wellbeing (EW). The RE scale asks the participant whether, in the last four weeks, he or she had had to reduce the time spent at work or on daily tasks because of feelings of sadness, depression, or nervousness and whether he or she had spent less time than wanted or planned on desired activities because of an emotional problem. The SF scale measures the ability of the participant to perform normal social activities without being hampered by physical or emotional issues. This scale explored whether and to what extent a participant’s physical health and/or emotional problems hindered or interfered with (in the last four weeks) social activities with family, friends, or other people. The EW scale determines whether the participant felt peaceful, happy, and calm in the past four weeks.

We considered a participant to be nonadherent to his/her medication when he/she reported forgetting or not taking that medication and/or having problems taking it. Pharmacy records were requested to verify adherence to medication. Two graduate students in clinical psychology administered the pre- and post-measures. In the first twelve cases, the agreement between raters was assessed. The same tests were administered one week before the beginning of the intervention and one week after the psychosocial intervention had concluded. The purpose of these tests was to determine whether there were changes that could be associated with the psychosocial intervention. Treatment acceptability measures included a satisfaction questionnaire designed for this program. The adherence-to-therapy techniques used by the therapist administering the intervention were monitored using videotapes or audiotapes of the sessions.

Three groups were developed for the intervention. Two of them were at the ASSMCA facilities in Trujillo Alto and San Patricio, while one was at Albizu University in San Juan.

We calculated the statistics using SAS and SPSS. McNemar’s test was used to assess the difference on a dichotomous dependent variable (adherence/nonadherence to treatment), and a paired-samples *t*-test was used for continuous dependent variables. Statistical significance was set at α = 0.05.

#### 2.2.2. Therapists Recruited and Assessment of Fidelity

Two graduate students from Albizu University were trained in the psychosocial intervention program (G.A. and Y.A.), and two other graduate students supported the participants during the different activities. In other words, each therapist had a co-therapist. Two graduate students in clinical psychology administered the pre- and post-measures (W.O. and L.A.). An agreement between raters was sought for the 12 cases (for both the YMRS and the MADRS). Weekly supervisory meetings were carried out with the purpose of following-up on the group interventions, including determining whether the therapists had adhered to the manual. After each session with the group, the principal investigator (P.I.) met with the therapists to review the recordings and to offer feedback. P.I. has vast clinical experience conducting cognitive behavioral therapy and motivational interviewing sessions. Both conceptual models were used as part of this intervention in addition to family psychoeducation and symptom management.

The two graduate students (G.A. and Y.A.) followed the therapist manual that had been developed in the first stage.

#### 2.2.3. Analysis

The feasibility and acceptability of the psychosocial intervention were assessed based on the participants’ retention, their acceptance of the new intervention, the researcher’s ability to recruit the target population, the viability of delivering the intervention, the participants’ satisfaction with the treatment, changes in adherence to treatment medication, and clinically significant changes in patient outcomes.

## 3. Results

Sociodemographic data and psychiatric diagnoses are included in Table 2.

The medical comorbidities and lifestyle characteristics of the sample that are shown in Table 3 were measured.

### 3.1. Feasibility and Acceptability

#### 3.1.1. Retention

Participants were recruited and consented on the same day as the initial orientation. A total of 24 participants began the intervention and were divided into three groups. Of the original participants, 71% completed the 12 intervention sessions (with the pre- and post-measures). This completion rate is comparable to those of other intervention studies requiring weekly participation [22]. Seven participants (five females and two males) could not complete all the interventions: six of the participants had transportation difficulties, and one had been discharged from the transitional home. We contacted the participants, who were then able to take the post-measure, allowing us to determine whether there were any changes from the pre-measure. Only three of the seven participants completed the post-measure.

#### 3.1.2. Clinically Significant Changes in the Patient Outcomes

Our primary clinical outcome was that BD patients at risk of CVD showed significant improvements in terms of their adherence to psychiatric and CVD medications, based on pre- and post-intervention measures. We took into consideration only those participants who completed all 12 sessions. Using a composite of the three questions mentioned in the procedures section, we determined (based on an analysis of the responses of the participants before and after the intervention) who had adherent behaviors and who had nonadherent behaviors. When reviewing the pharmacy records of the participants, we observed, and the participants confirmed that they would often buy all of the prescribed medication but not use all of it. For that reason, we were unable to use the pharmacy records as measures of adherence or nonadherence to treatment. Another issue that we detected had to do with the different medications of the participants from the transitional home in Trujillo Alto. In that home, all the medication was administered directly by a nurse, and for the most part, the patients felt themselves to be adherent. However, after interviewing the nurse, we discovered that while a given patient might have received his or her medication from that healthcare worker, that was no guarantee that he or she actually took the medication. That being the case, perceived adherence was higher in the patients than actual adherence.

We carried out McNemar’s test. We also obtained preliminary effect sizes related to pre-post changes on measures of self-reported adherence to medication. At baseline, 29% of the participants reported recent adherence to psychiatric medications; following the intervention, 82% reported being adherent. An exact McNemar’s test determined that the difference in the proportion of adherence pre- and post-intervention was statistically significant (*p* = 0.004) and had a large effect size (*d* = 0.82) (Table 4). Thus, we conclude that the psychosocial intervention program made a difference in the results in favor of adherence to treatment.

A paired-samples *t*-test for continuous dependent variables was used for the analysis of pre- and post-measures of attitudes toward medication through the DAI-10. This inventory has 10 items whose scores range from –10 to +10. A total score >0 is indicative of a positive attitude toward medication. A total score <0 is indicative of a negative attitude. Compared to the baseline measure (*M* = 4.0; *SD* = 4.4), the scores of the participants after the twelfth week of the psychosocial intervention were lower (*M* = 7.3; *SD* = 3.1), showing that the negative attitudes of the participants toward taking medication had lessened.

There was a significant difference in pre- and post-intervention: *t*(16) = −3.78; *p* = 0.002; *d* = 0.92. This is a significant change, improving the attitudes toward medication after the psychosocial intervention (as evidenced by the large effect size).

Our secondary outcome was that, according to the pre- and post-treatment measures, the targeted psychosocial intervention program was associated with significant improvements to the manic and depressive symptomatology and the health behaviors. The Spanish version of the YMRS is a clinical interview scale used in research to assess the severity of manic states. A paired-samples *t*-test was used for the pre- and post-intervention measures. The participants showed improvements in the severity of symptoms (decreasing manic symptoms) after the psychosocial intervention program (*M* = 4.5; *SD* = 2.8) compared to the baseline measure (*M* = 7.4; *SD* = 5.6). There was a significant change in pre- and post-measures (*t*(16) = 2.2; *p* = 0.046), with a medium effect (*d* = 0.52).

The Spanish version of the MADRS was used to take pre- and post-measures. This scale is used to measure the severity of depressive episodes in patients with mood disorders. We used a paired-samples *t*-test to assess the differences in depression symptomatology. Although we found an improvement in depression scores between the participants after the psychosocial intervention program (*M* = 16.4; *SD* = 13.1), compared to the baseline measures (*M* = 18.9; *SD* = 13.1), this improvement was not statistically significant—*t*(16) = 1.0; *p* = 0.324—and had a small effect (*d* = 0.24). Pre-intervention, 13% of the sample was euthymic; post-intervention, the proportion increased to 18%.

Cohen’s *k* was run for 12 of the YMRS and MADRS scales to determine whether there was agreement between the two independent raters; there was a very good agreement between them: k = 1.00; *p* = 0.000.

For the SF-36 Scales, there were no significant results; however, we still were able to obtain information about the participants via the pre- and post-measures. For example, in the RP and BP scales, we observed a decrease (greater limitations and limiting pain) due to physical health after finishing the intervention. On the mental component of health status, in the RE scale, the participants showed improvements related to work or other daily activities that could affect their emotional status. With regard to the SF scale, we observed improvements after the psychosocial intervention program. The scoring indicated a medium effect size (0.49) for this component, with a *p*-value of 0.062 (nonsignificant statistically).

#### 3.1.3. Treatment Satisfaction

As part of the post-intervention feedback, a satisfaction questionnaire was administered one week after the psychosocial intervention program was completed. The questionnaire was divided into four parts. The first seven questions explored each participant’s level of satisfaction with several components of the intervention and with the therapists, and they (the questions) explored how the intervention helped them (the participants) to improve their adherence to medication. For each question, each participant chose a number from 1 (not satisfied at all) to 10 (very satisfied). All the participants (100%) indicated that they were very satisfied with the therapists in charge of the sessions and with the topics that were covered in the various sessions. In terms of the entire intervention, 94% indicated that they were very satisfied, and 6% indicated that they were moderately satisfied. One hundred percent of the participants stated that this psychosocial intervention helped them to manage their adherence to the medication regimens required by their mental conditions. In addition, 82% of the participants indicated that the intervention helped them very much in terms of their adhering to the medications they took for their physical illnesses, while 12% expressed that this help was moderate. One person did not respond to this item. Related to healthy behaviors, 94% of the participants thought that the intervention helped them very much and 6% moderately. Finally, related to the management of the symptomatology of BD, 94% of the participants considered that the intervention was of much help, and 6% felt that it was moderately helpful.

On the second part of the questionnaire, the participants answered four questions. The questions asked participants to relate the most and least helpful aspects of the intervention, to make suggestions on how to improve the intervention manual, and to add any points or items that they felt should be addressed and/or discussed.

According to the content analysis of the questions asked in this section, the majority of the participants agreed that the intervention helped them to gain awareness of the importance of the medications that they took for their mental and physical illnesses (and of its being taken every day), of the need to accept their diseases, and of the need to manage their moods. Other issues that emerged were related to receiving help through support groups, having a therapist, and making changes in their lifestyles that would promote healthy behaviors. Psychoeducation and help for the management of stigma in the family context was a topic mentioned on one occasion.

For the second question, which explored the least helpful aspect of the intervention, the majority of the participants said that they could not identify any aspect that was not useful to them and that everything helped them. Two persons mentioned that the topic related to the management of smoking did not apply to them and so was the least useful. One person wrote that the topic related to the relationship between the service provider and the patient was repetitive. Another said that the time for the intervention was too short. A third person complained that sometimes, the people with more marked symptoms (of BD) talked so much that it was hard to concentrate.

The third question asked for suggestions on how to make the psychosocial intervention manual more useful; the majority of the participants responded with ideas about sharing (disseminating) it, offering such reasons for doing so as “it is vitally important to know the meaning of health conditions and alternatives treatments”; “the manual was well prepared”; “everything was fine.” There were other comments as well in the form of suggestions: that the testimonies of the participants be used to help other individuals, that stigma be emphasized more (in the intervention), that the importance of exercise be emphasized more (in the intervention), that individual interventions be included, and that the intervention differentiate between the diagnosis of BD and depression.

For the last question (regarding what the patients would like to see added or discussed), most of the participants said that they would not add anything else, but several opined that the following topics should be added: relapse management, what to do when you run out of medication, sexuality, studying and working, and self-motivation. One person indicated that it would be helpful to get the information (communicated via the intervention) in writing. One of the people suggested developing a symposium on bipolarity (it should be noted that no such symposium exists in Puerto Rico at this time) in order to educate the community on avoiding using the term “bipolar” in a pejorative way.

The third part of the questionnaire was aimed at determining whether the participants had any difficulty understanding the presentations or the information disseminated in the different sessions and whether the activities during the psychosocial intervention program were difficult. This part also had a question about enjoying the process. Sixty-five percent of the sample reported that the presentation was “not at all” difficult to understand; 18% said it was a little difficult, 12%, somewhat difficult, and 6% (one person), quite difficult.

Regarding the question about how difficult it was to understand the information contained in the manual, 77% informed us that it was not at all difficult; 12% said that it was a little difficult and 12% that it was somewhat difficulty. With respect to the activities, 71% stated that the activities were not at all difficult, 18% said that they were a little difficult, and 12% said that they were somewhat difficult. Finally, when asked whether they enjoyed the experience, 71% of the participants agreed that they enjoyed it a great deal and 30% that they enjoyed it fairly well.

The fourth and last part of the questionnaire covered the manual that we developed for the intervention. The participants responded to five items using a five-point Likert scale (totally agree, agree, neither agree nor disagree, disagree, and totally disagree). For item one, 77% of the participants totally agreed, and 12% agreed that the intervention would be helpful to other persons; two of the participants chose not to answer. When asked whether the psychosocial intervention was specifically helpful to them, 65% totally agreed that it was, 24% agreed that it was, and 6% disagreed that it was helpful. When asked whether they would participate in another group intervention to improve adherence to treatment, 71% of the group totally agreed that they would, 6% agreed that they would, 6% disagreed that they would, and 6% totally disagreed that they would. Of those who totally agreed that they would participate in an intervention aimed at improving their mental and physical health, 65% further totally agreed that they would do so, 12% agreed that they would do so, 6% were neutral on the subject, and 12% were in total disagreement with participating in such a group. The final item of the five items explored whether the participants would have preferred that the intervention be carried out with individuals rather than a group. In this one, 47% were in total agreement that they would, 6% agreed that they would, and 35% were neutral on the subject.

#### 3.1.4. Other Measures: Cognitive Functioning

As stated earlier, we used the MMSE-2 to rule out cases of severe cognitive deterioration and dementia as the cause or causes of any changes that we saw. There was variability related to the MMSE-2 before and after the psychosocial intervention. We used the MMSE-2 with a cut-off point of ≥ 24 (normal). According to the MMSE-2, 82% of our participants had normal cognitive functioning at the beginning of the psychosocial intervention program (*M* = 24.8; *SD* = 2.6). After the intervention, 59% had normal cognitive functioning (*M* = 24.7; *SD* = 3.5). The rest of the cases (41%) had scores that indicated mild cognitive deterioration. A case-by-case analysis showed that 12% of the participants that completed the psychosocial intervention presented the same score on pre- and post-measures (normal functioning). Of the participants (47%) whose scores changed, 29% saw their scores decrease from one to three points on the MMSE-2. Of the 41% of participants whose scores increased after the intervention, one showed a significant increase of five points on the MMSE-2. Of the participants with decreasing scores, three of them were from 53 to 58 years old, and two of them were from 38 to 47 years old.

Patients with BD tend to experience cognitive deterioration [23]; this is characteristic of this population and is highly predictive of poor psychosocial function and lower quality of life [24]. Debate exists about the onset and progression of the cognitive impairment in BD. Cross-sectional studies on BD patients suggest that these impairments follow a deteriorating course from initial onset to chronicity. It is well known that during euthymic episodes, BD patients experience cognitive impairments [25]. Cognitive impairment may be influenced by several confounding variables, which include residual symptoms, the severity of the psychiatric disorder, lifestyle factors, medication side effects, and electroconvulsive therapy (ECT). In our sample, we found that 29% of the participants had an history of ECT. All of these participants had normal scores on the MMSE-2 before the intervention. However, after the intervention, 41% of them showed mild cognitive impairments. There is a debate about the effect of ECT on cognition of bipolar patients. Several studies have established an association between ECT, and cognitive impairment related to the electrode placement [26], while others suggest that there is no cognitive impairment with the use of ECT [27]. Educational or occupational achievements can help to improve the cognitive outcomes of BD patients. However, regarding education as a confounding variable with respect to the MMSE-2, in this pilot study, we found that with or without their having a high education level, people with BD showed mild cognitive deterioration.

#### 3.1.5. The Family Session

A single psychoeducative session was carried out with family members of the participants; the session discussed the BD diagnosis, adherence problems, and the need for familial support. However, it also was an opportunity for those family members that attended to talk about their own needs. Only 24% of the family members participated. From the beginning, each participant was aware that one session with a family member would be carried out as part of the intervention. However, when it was announced that it was time for that session, some of the participants decided that they did not want to have anyone from their families participate. In addition, some relatives, though showing an interest, were unable to attend the session. The issue of family is very important since these people tend to burn out in the process of dealing with a BD patient. As was indicated in a previous study [12], some patients feel that their poor adherence to treatment is influenced by their close relatives’ perceptions of which illnesses require medication and which do not.

## 4. Discussion

We pilot tested a previously developed psychosocial intervention manual on a Puerto Rican sample of Spanish-speaking patients with BD who were at risk of CVD. We established the first stage (stage Ia and stage Ib) of a behavioral therapy trial to develop an intervention for nonadherent bipolar patients and then, after having developed it, pilot tested it. In Stage Ia, we developed a psychosocial intervention manual that was designed to address culturally sensitive elements in our Hispanic population. This intervention consisted of twelve sessions of group therapy, including a session in which several patients brought a family member or his/her significant other. The four approaches that we used were based on motivational interviewing strategies, cognitive-behavioral techniques, family psychoeducation, and symptom management. In Stage Ib, which consisted of an open, single-group design and in which each subject was his/her own control, 24 BD patients began the psychosocial intervention. Of them, 17 completed the 12 sessions. The original 24 patients were divided into three groups of eight participants, each. We took several measures, pre- and post-intervention, to determine the feasibility and acceptability of the intervention and to ascertain any improvements in the adherence behaviors of these patients. 

We confirmed our primary outcome (significant improvements in adherence to psychiatric medication and that of physical comorbidities) with our analysis of the adherence self-reports (a large effect size was observed). We determined the changes (also significant) in medication attitude through the DAI-10 (a large effect size was observed here as well). 

Our secondary outcome, elicited through the pre- and post-treatment measures, was that there were significant improvements in both the manic and depressive symptomatology and the health behaviors. We obtained statistically significant results on the YMRS, in which patients showed decreases in mania symptoms (with medium effect size). The results of the MADRS were not statistically significant, but we observed decreases in depression symptoms, with a small effect size. Regarding health behaviors, we did not obtain statistically significant results in the SF-36 (self-report). However, we observed some changes when we took a closer look at the three SF-36 scales (those related to the mental health component), which assessed limitation(s) due to emotional problems, social functioning, and emotional wellbeing. Improvements were seen in the performance of daily activities (i.e., that a given participant did not feel sad or depressed or have any kind of emotional problem that prevented his or her performance of such activities). After the psychosocial intervention program, we observed improvements in the performance of social activities with family, friends, and other significant people without interferences caused by physical or emotional problems. This scale showed a medium effect size of 0.49. For the EW scale, no statistically significant results were obtained, only tiny increases related to feelings of peace, happiness, and calm (experienced in the four weeks prior to the intervention). Interestingly, in two of the three scales associated with physical health status, we observed decreases in (or more limitations to) the ability to perform physical work because of physical health issues or limiting pain. No statistically significant results were obtained. 

As was revealed earlier, before and after the psychosocial intervention, we used the MMSE-2 to determine whether there was any severe cognitive deterioration and/or dementia. Cognitive deterioration is common in bipolar patients [28]. Our results indicated greater levels of mild cognitive deterioration in some of the participants after the intervention. We think that this could be related to several confounding variables that are linked to the severity or degree of a given individual’s psychiatric disturbance, his or her social and cultural contexts and previous abilities, and both the number of years that he or she has had the diagnosis and the types of medication that he or she is taking. We found that ECT had been a treatment for 29% of the BD sample before their participation in the study, and of them, 41% showed mild cognitive impairment (according to the MMSE-2) after the intervention. There is debate as to whether ECT leads to cognitive impairment or not. Additionally, the literature maintains that patients with BD show cognitive impairment that persists in remission periods and significantly influences those patients’ psychosocial outcomes, with a large degree of heterogeneity among them [29].

Finally, 100% of the patients confirmed that the psychosocial intervention program helped them to manage their adherence to the medication prescribed for their psychiatric conditions. In addition, 100% of the participants said that they were very satisfied with the therapists in charge of the sessions and with the topics that were covered in each session. Interestingly, participant satisfaction with the intervention’s ability to improve adherence to psychiatric vs. physical illness medications differed from the previous in that it was generally lower. While 82% of the participants indicated that the intervention was greatly helpful to them in increasing their adherence to their medications for physical illnesses, 12% revealed that the intervention was only moderately helpful. These results are excellent and are consistent with the goal of this study, but it should be noted that the difference between adherence to psychiatric medication and to medication for physical illnesses—which is an important difference—was addressed in a previous study by the main author [13]; that study revealed that the pattern of nonadherence to psychiatric medications is different from that of nonadherence to medications used in the treatment of physical illnesses. In terms of healthy behaviors, 94% of the participants thought that the intervention helped them a great deal, while 6% felt that it helped them moderately. Finally, in terms of the management of the symptomatology of BD, 94% of the participants considered the intervention to have been of great help, and 6% felt that it was of moderate help.

The results of the satisfaction questionnaire are consistent with the information obtained by the SF-36 scales. The overall increase in adherence to treatment for psychiatric conditions can be associated with the responses of the patients on the mental health component of the SF-36, in which improvements were seen.

Using the results obtained by the open trial, changes to the manual will be made. These changes will incorporate both the previously mentioned results and the recommendations of the participants. Each topic will be reviewed and the language level (of the manual) checked to ensure that people with a middle-school (at minimum) education and/or mild cognitive impairment will be able to understand the directions.

The study had several limitations. The first is that it had a small sample size; a study with larger sample size and a control group should be undertaken. Next, there was a low representation of males. Following that, the participants were not formally assessed to confirm the psychiatric diagnosis of BD or of any psychiatric comorbidity. Continuing, the presence of CVD risk factors (physical illnesses) was determined by self-report. The pharmacy records were included with the purpose of corroborating the use of medications and were assessed to determine any changes in (a given patient’s) adherence to (his/her) medication. However, though the majority of participants indicated that they ordered all the medications they were prescribed, their having done so does not mean that they took all of them. These patients had medical insurance from the government, and the prescriptions were cost-free. Accordingly, we had to discard the participants’ pharmacy records as an objective measure: the cognitive functioning of the participants may have been influenced by one or both of the factors mentioned previously. Finally, the participants in this study were not representative of the entire population of bipolar patients in PR. 

Despite the limitations, we tested the b component of the first stage of our previously developed intervention intended to promote treatment adherence in BD patients at risk of CVD, with significant improving outcomes. The participants felt that the intervention helped them to improve their medication adherence behaviors for both their mental and their physical illnesses.

## 5. Conclusions

This manuscript describes a culturally sensitive psychosocial intervention program (with a manual in Spanish) for BD patients at risk of CVD and was designed following the SMBT; the intervention was developed in a previously completed study (stage Ia) and pilot tested in the study described herein (stage Ib). The intervention helped to increase treatment adherence and therefore improved the treatment outcomes of the intervention participants by decreasing manic and depressive symptomatology. Further research will be needed, with such changes to the original protocol as are necessary to refine this intervention in preparation for stage II in the series of studies.

We conclude that the improvement registered in each post measure of the scales used could be associated with the improvements of the adherence to treatment that were experienced during the psychosocial intervention program. Our intervention had a positive impact on the bipolar patients who participated in it, leading to reductions in their psychiatric symptoms and improvements in their CVD-risk-associated physical illnesses. Our results are consistent with those of other studies, in which better results are seen in minority groups when a culturally adapted intervention is used [16]. This culturally sensitive psychosocial intervention program improved outcomes in comparison to interventions that did not use, create, or adapt interventions for the culture of their clients.

## Figures and Tables

**Table 1:** Psychosocial Intervention Manual.

Contents	
Part I. Strategies for Adherence	Session 1: Identifying Barriers
	Session 2: Bipolar Disorder: Distinction between myself, my symptoms, and stigma management
	Session 3: Identifying the Goals for Treatment Adherence
	Session 4: Effective Medication Management
	Session 5: The Service Provider: My psychiatrist and how to take responsibility for my treatment in conjunction with that treatment
	Session 6: The Influence of Family and/or Significant Others
Part II. Developing Healthy Behaviors	
	Session 7: Complementary Behavioral Activities to Promote Physical and Mental Health: Eating healthy
	Session 8: Complementary Behavioral Activities to Promote Physical and Mental Health: Physical Exercise and Related Activities
	Session 9: Complementary Behavioral Activities to Promote Physical and Mental Health: Managing cigarette use
	Session 10: Complementary Behavioral Activities to Promote Physical and Mental Health: Sleep management
Part III. Interpersonal Relationships and Support Groups	
	Session 11: Encouraging the Development of Networks and Groups: Social support–identifying support candidates
	Session 12: The Social Support Group for Nonadherent Behaviors

**Table 2:** ^1^ Sociodemographic data and psychiatric diagnoses of the 24 participants.

Variable	M/F *f (%)*	Mean	SD
Age in years		44.21	9.23
Gender	2 (8)/22 (92)		
Diagnosis			
Bipolar disorder I	1 (50)/7 (32)		
Bipolar disorder II	0 (0)/11 (50)		
Bipolar disorder unspecified	1 (50)/4 (18)		
Ethnicity			
Puerto Rican	2 (100)/21 (96)		
Dominican	0 (0)/1 (5)		
Education			
High school	0 (0)/4 (18)		
Technical certificate	0 (0)/5 (23)		
Associate degree	1 (50)/3 (14)		
Bachelor’s degree	1 (50)/5 (23)		
Master’s degree	0 (0)/4 (18)		
Professional certification	0 (0)/1 (5)		
Marital Status			
Single	2 (100)/10 (46)		
Married	0 (0)/2 (9)		
Divorced	0 (0)/7 (32)		
Separated	0 (0)/0 (0)		
Widowed	0 (0)/2 (9)		
In a consensual union	0 (0)/1 (5)		
Number of Children		1.38	1.2
0	2 (100)/5 (23)		
1	0 (0)/5 (23)		
2	0 (0)/10 (46)		
3	0 (0)/1 (5)		
5	0 (0)/1 (5)		
Number of Family Members		2.75	1.92
Alone	1 (50)/7 (32)		
2	0 (0)/4 (18)		
3	1 (50)/5 (23)		
4	0 (0)/2 (9)		
5	0 (0)/3 (14)		
9	0 (0)/1 (5)		
Residence			
Own	0 (0)/4 (18)		
Rent	1 (50)/6 (27)		
Stay with another family	0 (0)/6 (27)		
Transitional home	1 (50)/6 (27)		
Annual income			
$0–$999	1 (50)/2 (9)		
$1000–$2499	0 (0)/5 (23)		
$2500–$4999	0 (0)/5 (23)		
$5000–$7499	0 (0)/1 (5)		
$7500–$9999	1 (50)/3 (14)		
$10,000–$14,999	0 (0)/1 (5)		
$15,000–$24,999	0 (0)/3 (14)		

^1^ Descriptive statistics (frequencies and percentage) were used for categorical data and mean and SD for continuous variables.

**Table 3:** Medical comorbidities and lifestyle characteristics of the 24 participants.

Variable		Mean	SD
Medical comorbidities			
	*f* (%)	BMI 30.4	5.3
Obese (BMI ≥ 30 kg/m^2^)	12 (50)		
Overweight (25–29.9)	8 (33)		
Hypertension	11 (46)		
Diabetes	4 (17)		
Hypoglycemia	1 (4)		
High cholesterol	6 (25)		
Lifestyle Behaviors			
Smokes	4 (17)		
High level of stress	22 (92)		
Diet *	Pre		Post
Unhealthy	11 (65)		7 (41)
Healthy	6 (35)		10 (59)
Exercise *	6 (35)		8 (47)

* In these rows, we are including the pre- and post-measures.

**Table 4:** Pre- and post-treatment scores of those who completed the intervention.

	Pre-Intervention *n* = 17	Post-Intervention *n* = 17	*p*-Value	Effect Size *d*
	*M (SD)*	*M (SD)*		
Self-report adherence	4.2 (4.6) 29%	7.3 (3.1) 82%	0.004	0.82
Drug Attitude Inventory (DAI-10)	4.0 (4.4)	7.3 (3.1)	0.002	0.92
Young Mania Rating Scale	7.4 (5.6)	4.5 (2.8)	0.046	0.52
Montgomery—Åsberg Depression Rating Scale	18.9 (13.1)	16.4 (13.1)	0.324	0.24
*SF-36 Scales*				
Physical functioning	57.7 (35.5)	57.9 (39.8)	0.951	0.01
Role limitations due to physical health	33.8 (39.5)	19.1 (33.7)	0.154	0.36
Role limitations due to emotional problems	45.1 (42.4)	51.1 (41.1)	0.554	0.15
Energy/fatigue	49.7 (24.0)	46.8 (26.6)	0.492	0.17
Emotional wellbeing	54.6 (27.9)	55.8 (26.6)	0.827	0.06
Social functioning	36.0 (25.3)	50.1 (38.1)	0.062	0.49
Bodily pain	41.2 (34.4)	30.9 (28.1)	0.159	0.36
General health	53.9 (28.8)	52.0 (26.5)	0.641	0.12
Health change	2.2 (1.0)	2.3 (1.3)	0.826	0.09
Mini-Mental State Examination-2	24.8 (2.6) 82% (≥24)	24.7 (3.5) 59% (≥24)	0.857	0.04

## Data Availability

Data are contained within the article.
